# Evaluation of intrarenal vein flow patterns during routine echocardiography

**DOI:** 10.1007/s00380-025-02523-9

**Published:** 2025-02-04

**Authors:** Tomoo Nagai, Hitomi Horinouchi, Tabata Hirotsugu, Yuji Ikari

**Affiliations:** 1 Division of Cardiology, Department of Internal Medicine, Tokai University School of Medicine, Shimokasuya 143, Isehara-shi, Kanagawa 259-1193 Japan; 2 Department of Cardiovascular Medicine, Federation of National Public Service Personnel Mutual Aid Associations, Mishuku Hospital, Kamimeguro 5-33-12, Meguro-ku, Tokyo 153-0051 Japan

**Keywords:** Intrarenal vein flow, Echocardiography, Diagnosis, Heart failure

## Abstract

**Objective:**

Intrarenal vein flow (IRVF) abnormalities can predict cardiovascular events including heart failure. This study aimed to evaluate the utility of short IRVF scans during routine comprehensive transthoracic echocardiography (TTE) examinations in a standard TTE laboratory.

**Methods:**

We screened consecutive patients who underwent elective TTE at our Ultrasound Imaging Laboratory between March 2018 and July 2019 and prospectively enrolled those who completed a 5 min IRVF scan during the 30 min TTE procedure.

**Results:**

Among the 2101 screened patients, 1326 were included in the study cohort (age: 73 ± 13 years, 756 men). IRVF abnormalities were detected in 13 (1.0%) patients. Twenty-one cardiac events were observed (1.6%, 21/1326): one myocardial infarction and 20 heart failures. Cumulative survival probability plots were generated using the Kaplan–Meier method within 6 months after the TTE index day and assessed using the log-rank test. The plots revealed significantly worse prognoses in patients with elevated right arterial pressure (RAP) and abnormal IRVF, when compared to normal RAP or normal IEVF (*p* < 0.0001 and *p* < 0.0001, respectively). In a receiver operating curve analysis to predict the occurrence of cardiovascular events, E/e’ had moderate predictive potential (area under the curve: 0.795, *p* < 0.0001), and the combination of E/e’ and IRVF abnormality had better predictive potential than did E/e’ alone (*p* = 0.043).

**Conclusion:**

Although rarely observed on TTE, IRVF abnormalities improve the ability of E/e’ to detect cardiac events, especially heart failure. Further large-scale prospective studies are required to confirm our findings.

## Introduction

Increased central venous pressure (CVP) (nearly equal to right atrial pressure [RAP]) causes systemic congestion, one of the major signs of heart failure, and worsens chronic compensated and relapsed decompensated heart failure through cardiorenal association [1, 2]. Although CVP is a useful predictor of clinical outcomes in patients with heart failure [3], current methods of measuring the inferior vena cava (IVC) diameter using transthoracic echocardiography (TTE) may yield different results [4].

The diagnostic utility of intrarenal vein flow (IRVF) patterns detected via renovascular ultrasound was first reported in patients with renal dysfunction or diabetes [5, 6]. This noninvasive technique was soon used to diagnose renal congestion in obstructive uropathy [7, 8] and, more recently, to manage cardiorenal syndrome, which is caused by high CVP [9]. IRVF imaging measures CVP; moreover, by uniquely visualizing IRVF abnormalities. Therefore, we hypothesized that comprehensive TTE would better predict cardiac events when accompanied by IRVF imaging. Thus, this study aimed to test this hypothesis by performing short IRVF scans during routine comprehensive TTE examinations in elective patients in a standard TTE laboratory.

## Methods

We screened consecutive patients who electively underwent comprehensive TTE between March 2018 and July 2019 at the Ultrasound Imaging Laboratory, Department of Laboratory Medicine, Federation of National Public Service Personnel Mutual Aid Associations, Mishuku Hospital (Meguro, Tokyo, Japan), and performed additional brief IRVF scans in the scheduled timeline of TTE study. The exclusion criterion was technically difficult case for appropriate IRVF scans within 5 min. This secondary care regional medical center has 200 beds and receives patients with cardiovascular emergencies under the jurisdiction of the local authorities and regional government. Owing to its limitations, there is no maintenance hemodialysis service for either in-patients or out-patients.

All elective comprehensive TTE examinations were performed by a single ultrasound imaging technician within a scheduled procedure time of 30 min. For TTE, an iE33 ultrasound imaging system (Philips Healthcare, Andover, MA, USA) with a S5-1 sector transducer was used. All comprehensive TTE procedures complied with current guidelines [10–14]. Left atrial volume and left ventricular volume were assessed using the modified biplane Simpson method. Valvular stenosis and regurgitation were evaluated and graded using a multiparametric approach, including qualitative, semiquantitative, and quantitative parameters, in accordance with current guidelines [13, 14]. RAP, used as a CVP surrogate, was estimated using the three-grade classification system recommended by Lang et al. [12]. In our study, conditions with more than mild valvular pathology were considered significant valvular diseases.

**Fig. 1:**
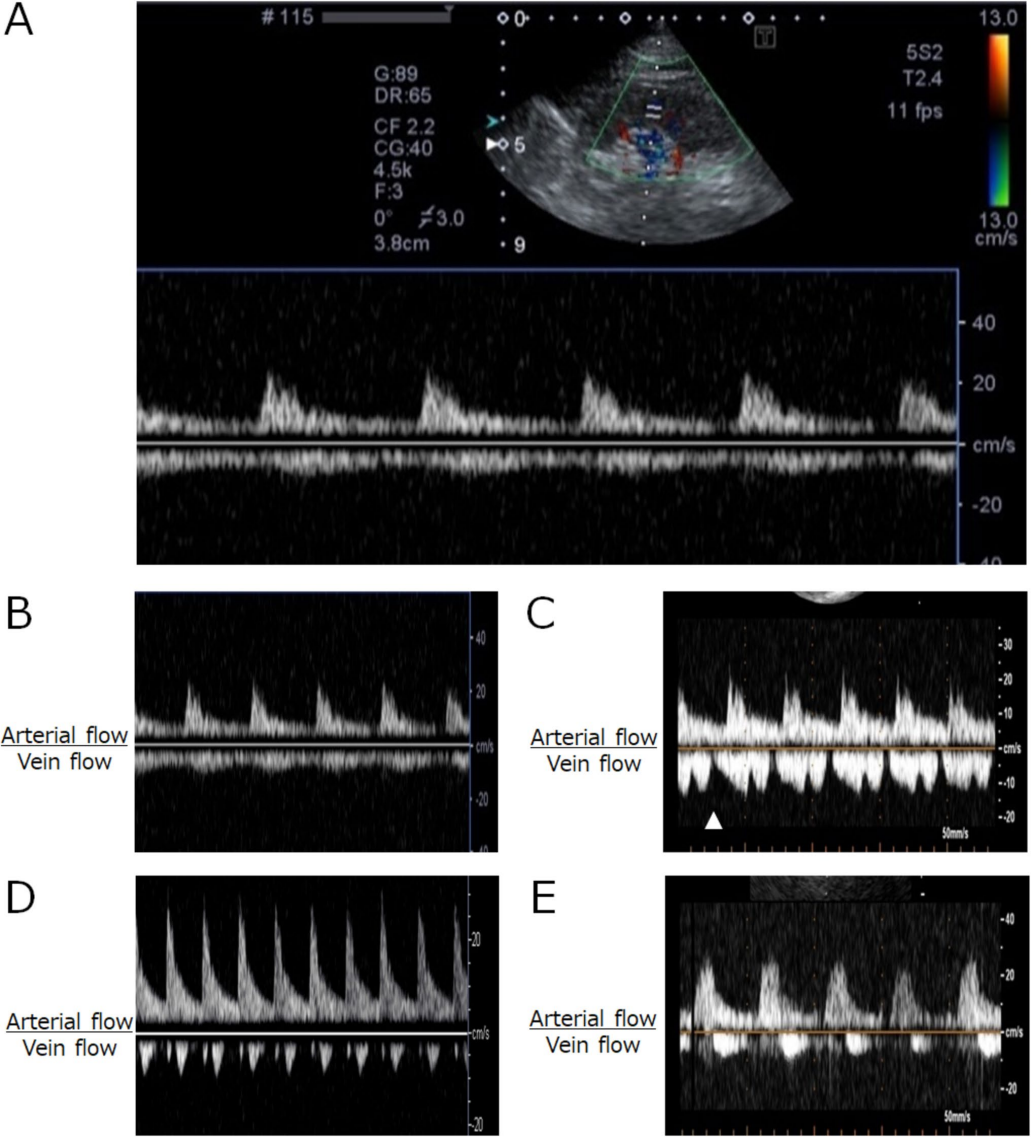
Patterns of intrarenal vein flow. **A**, a representative image of intrarenal vein flow; **B**, continuous flow (normal); **C**, discontinuous flow; **D**, biphasic flow (diastole and systole); **E**, monophasic flow (systole). The arrowhead in panel **C** indicates presystolic disruption

We consecutively acquired IRVF images at the end of the TTE examinations. The IRVF imaging process has been described previously [9]. However, we simplified this process to allow its completion within the TTE procedure time of 30 min. The same technician performed both procedures. For IRVF imaging, the targeted side of the kidney (preferably the right side) was scanned, with the patient in the same position (usually the left lateral decubitus position) and bed as for TTE; the same ultrasound probe used for both TTE and IRVF imaging. The maximum time for IRVF scanning was 5 min. As shown in Fig. 1A, the velocity range for color Doppler imaging was set to 13 cm/s. Color Doppler images were used to identify interlobar vessels, and the sample volume was based on the color Doppler signals derived from the interlobar arteries. Pulsed Doppler waveforms of the interlobar arteries and veins were recorded simultaneously. In all cases, the waveforms were recorded in more than three cardiac cycles.

All TTE and IRVF images acquired at our institute were transferred via an institutional network to the moving picture server of an ultrasound imaging laboratory (PrimeVitaPlus, Nihon Kohden Corp., Shinjyuku-ku, Tokyo, Japan). The TTE results were reviewed, and conventional measurements were performed in accordance with current guidelines [10–14] and interpreted by cardiologists. An experienced cardiologist classified the IRVF patterns on the IRVF images via visual assessment without numeric analysis: The four patterns were continuous (normal waveform, Fig. 1B), discontinuous (abnormal waveform, Fig. 1C), biphasic (abnormal waveform, Fig. 1D), and monophasic (abnormal waveform, Fig. 1E) [9]. Discontinuation of IRVF was defined as a pattern in which the velocity at the nadir was 0 cm/s.

We retrieved the following information from digitalized medical records: clinical baseline patient characteristics; laboratory data, including serum creatinine level and estimated glomerular filtration rate (eGFR); and clinical diagnosis. The observation period was 6 months after the TTE index day. Cardiac events were classified as myocardial infarction, heart failure (newly developed or relapsed), or cardiac-related death. This observational study was approved by the Institutional Review Board for Clinical Research of Mishuku Hospital. The study was conducted in accordance with the principles of the Declaration of Helsinki. The requirement for written informed consent was waived because of the low-risk nature of the study. An opt-out approach was employed.

Numerical data are presented as either mean ± standard deviation or median and range, or as percentages for categorical variables. Depending on normality, continuous variables were compared between two unpaired groups using the unpaired Student’s t test or Wilcoxon rank-sum test. The ratios of the category values between two groups were analyzed using the chi-square test. The agreement between the different test results was evaluated by Kappa statistics. Logistic regression was performed for multivariate analysis. We considered all parameters with a *p*-value < 0.05 as possible covariate candidates. Cumulative survival probability plots were generated using the Kaplan–Meier method, and the curves were compared using the log-rank test. The cutoff value was calculated using receiver operating characteristic (ROC) curve analysis. Area under the curve (AUC) comparisons were performed using the Delong test [15]. All statistical analyses were performed using JMP 14.0.0 (SAS Institute, Cary, NC, USA), and the significance level was set at *p* < 0.05.

## Results

A total of 2101 consecutive patients electively underwent standard comprehensive TTE during the study period. Among them, 775 (37%) were excluded according to the predefined criterion. Thus, our study comprised 1326 patients (age: 73 ± 13 years, 756 men), none of whom were undergoing hemodialysis. The background characteristics of the patients are presented in Table 1. IRVF abnormalities were detected in 13 (1.0%) patients: five had discontinuous waves, three had biphasic waves, and five had monophasic waves. We divided the study cohort into two groups based on the presence or absence of abnormal IRVF. Among the background factors, the frequency of dyslipidemia, and atrial fibrillation during TTE between these groups. Additionally, the frequencies of valvular heart disease (e.g., aortic, mitral, or tricuspid regurgitation) differed significantly between these groups.

**Table 1:** Background factors

	Total (*n* = 1326)	IRVF abnormality present (*n* = 13)	IRVF abnormality absent (*n* = 1313)	*p*-value
Sex, Male, *n* (%)	756 (57)	7 (54)	749 (57)	0.817
Age, year	73 ± 13	73 ± 17	73 ± 14	0.871
Height, cm	160 ± 11	156 ± 9	160 ± 11	0.189
Weight, Kg	59 ± 13	53 ± 11	59 ± 13	0.078
Body surface area, m^2^	1.60 ± 0.20	1.50 ± 0.19	1.61 ± 0.20	0.072
In-hospital patient, *n* (%)	276 (21)	5 (39)	271 (21)	0.115
Referral from cardiovascular physician, *n* (%)	695 (52)	5 (39)	690 (53)	0.311
Creatinine^a^, mg/dL	0.95 ± 0.43	1.01 ± 0.43	0.94 ± 0.43	0.647
eGFR^a^, mL/min/1.73 m^2^	60 ± 19	58 ± 18	60 ± 19	0.700
eGFR: < 45 mL/min/1.73 m^2^, *n* (%)	207 (16)	2 (15)	205 (16)	0.982
eGFR: < 15 mL/min/1.73 m^2^, *n* (%)	6 (0.5)	0 (0)	6 (0.5)	0.807
Hypertension, n (%)	142 (10)	1 (8)	141 (11)	0.927
Diabetes Miletus, n (%)	122 (9)	0 (0)	122 (9)	0.716
Dyslipidemia, *n* (%)	17 (1)	1 (8)	16 (1)	0.039
Atrial fibrillation at TTE, *n* (%)	173 (145)	7 (54)	166 (13)	0.001
Pacemaker implantation, *n* (%)	8 (0.6)	0 (0)	8 (0.6)	0.778
Coronary artery disease, *n* (%)	307 (23)	1 (8)	306 (23)	0.414
Arrythmia (except atrial fibrillation), *n* (%)	67 (5)	0 (0)	67 (5)	0.701
Cardiomyopathy, *n* (%)	65 (5)	0 (0)	65 (5)	0.709
Chronic heart failure, *n* (%)	67 (5)	2 (15)	65 (5)	0.087
Venus thromboembolism, *n* (%)	18 (1)	0 (0)	18 (1)	0.909
Chronic pulmonary disease, *n* (%)	35 (3)	0 (0)	35 (3)	0.833
Gastrointestinal disease, *n* (%)	55 (4)	0 (0)	55 (4)	0.748
Cerebral vascular disease, *n* (%)	184 (14)	3 (23)	181 (14)	0.626

Bold texts indicate statistically significante

*GFR* estimated glomerular filtration rate, *IRVF* intrarenal vein flow, *TTE* transthoracic echocardiography

^a^Excluding 188 cases (14%) with missing value

As presented in Supplemental Tables 1, 2, the patients excluded from our study were older than those included and had lower body weights and more referrals from non-cardiovascular physicians. Additionally, the excluded cohort contained more men, in-patients, technically difficult TTE cases, and missing TTE values (e.g., left ventricular ejection fraction [LVEF], E/e, and RAP). However, the frequency of missing TTE value in tricuspid regurgitation pressure gradient was not significantly different, nor were the frequencies of severe obesity (body mass index > 30 kg/m^2^), depressed LVEF (< 50%), or elevated RAP (≥ 15 mmHg).

**Table 2:** Echocardiographic parameters in patients with intrarenal vein flow abnormality

	Total (*n* = 1326)	IRVF abnormality present (*n* = 13)	IRVF abnormality absent (*n* = 1313)	*p*-value
Aortic stenosis: ≥ moderate, *n* (%)	21 (2)	0 (0)	21 (2)	0.646
Aortic regurgitation: ≥ moderate, *n* (%)	57 (4)	3 (23)	54 (4)	< 0.001
Mitral stenosis: ≥ moderate, *n* (%)	3 (0.2)	0 (0)	3 (0.2)	0.863
Mitral regurgitation: ≥ moderate, *n* (%)	86 (6)	4 (31)	82 (6)	< 0.001
Tricuspid regurgitation: ≥ moderate, *n* (%)	113 (9)	7 (54)	106 (8)	< 0.0001
Any of left-sided VHD: ≥ moderate, *n* (%)	138 (10)	6 (46)	132 (10)	< 0.0001
Aortic root diameter, mm	28 ± 5	29 ± 6	28 ± 5	0.756
Left atrial volume index, mL/m^2^	26 ± 24	53 ± 57	26 ± 27	< 0.0001
Left ventricular end-diastolic volume, ml	90 ± 34	97 ± 32	90 ± 34	0.453
Left ventricular ejection fraction, %	68 ± 12	66 ± 12	68 ± 12	0.458
Interventricular septum thickness, mm	9.7 ± 1.9	9.3 ± 1.6	9.7 ± 1.9	0.477
Posterior wall thickness, mm	9.9 ± 1.6	9.2 ± 0.4	9.9 ± 0.0	0.091
Left ventricular mass index, g/m^2^	91 ± 27	97 ± 38	91 ± 26	0.402
Relative wall thickness	0.46 ± 0.11	0.41 ± 0.08	0.46 ± 0.11	0.060
E wave velocity, cm/s	65 ± 21	98.1 ± 37.3	65.0 ± 21.0	< 0.0001
A wave velocity, cm/s	81 ± 21	87.6 ± 22.4	81.0 ± 21.3	0.453
e’ velocity, mm/s	6.2 ± 2.1	7.2 ± 3.8	6.2 ± 2.0	0.086
E/e’	11.4 ± 4.6	17.5 ± 14.0	11.3 ± 4.4	< 0.0001
Right atrial pressure: ≥ 15 mmHg, *n* (%)	11 (0.8)	4 (31)	7 (0.5)	< 0.0001

*IRVF* intrarenal vein flow, *VHD* valvular heart disease

Bold texts are indicating statistically significant

The echocardiographic features of the study cohort are listed in Table 2. We excluded parameters with > 20% missing values, such as the tricuspid regurgitation pressure gradient, when comparing patients with and without IRVF abnormalities. We found that left atrial volume, E wave velocity, E/e’, and percentage of patients with elevated RAP were larger in the abnormal vs. normal group (53 ± 57 vs. 26 ± 27 mL/m^2^, *p* < 0.0001; 98.1 ± 37.3 vs. 65.0 ± 21.0 cm/s, *p* < 0.0001; and 17.5 ± 14.0 vs. 11.3 ± 4.4, *p* < 0.0001; 31% vs. 0.5%, *p* < 0.0001, respectively). No other factors differed significantly between these groups.

The correlations between IRVF abnormalities and RAP are presented in Table 3. Nine of the 13 (69%) patients with abnormal IRVF had non-elevated RAP (< 15 mmHg), and seven of 11 (64%) patients with elevated RAP (≥ 15 mmHg) had normal IRVF. Thus, in total, 16 patients had discrepant IRVF and RAP results. The kappa statistics between IRVF and RAP results was 0.99 ([1326–16]/1326). Comparison of patients with abnormal IRVF and non-elevated RAP with patients without this combination revealed no significant differences in the background factors, including serum creatinine level, eGFR, and frequency of eGFR < 45 mL/min/1.73 m^2^ (1.07 ± 0.46 vs. 0.95 ± 0.43 mg/dL, *p* = 0.435; 52.3 ± 17.0 vs. 60.2 ± 18.6 mL/min/1.73 m^2^ *p* = 0.230; and 22.2% vs. 15.6, *p* = 0.584, respectively). No patients with IRVF abnormalities and non-elevated RAP had an eGFR < 15 mL/min/1.73 m^2^.

**Table 3:** Right atrial pressure and intrarenal vein flow abnormality

	Intrarenal vein flow abnormality			
	Subtotal %	Discontinuous wave	Biphasic wave	Monophasic wave
Right atrial pressure: 3 mmHg (*n* = 1281)	5 (0.4)	2	1	2
Right atrial pressure: 8 mmHg (*n* = 29)	4 (14)	2	1	1
Right atrial pressure: 15 mmHg (*n *= 11)	4 (36)	1	1	2
Total (*n* = 1326)	13 (1)	5	3	5

Twenty-one cardiac events were observed (1.6%, 21/1326): one myocardial infarction and 20 heart failures, either de novo or relapsed. Cardiac-related death occurred in four patients. Table 4 lists the echocardiographic parameters associated with the cardiac events, as determined via linear regression analysis. In model 1, where elevated RAP was one of the covariates, more than mild tricuspid regurgitation, left ventricular mass index, E/e’, and elevated RAP predicted cardiac events (odds ratio [OR]: 5.248, 95% confidence interval CI 1.830–14.971, *p* = 0.004; OR: 1.022, 95% CI 1.009–1.036, *p* = 0.002; OR: 1.137, 95% CI 1.069–1.209, *p* < 0.0001; OR: 18.930, 95% CI 4.104–87.319, *p* < 0.001, respectively). In model 2, where IRVF abnormality was one of the covariates, more than mild tricuspid regurgitation, left ventricular mass index, E/e’, and IRVF abnormality predicted cardiac events (OR: 5.245, 95% CI 1.732–1.5.797, *p* = 0.006; OR: 1.023, 95% CI 1.008–1.037, *p* = 0.001; OR: 1.131, 95% CI 1.053–1.216, *p* < 0.001; OR: 74.233, 95% CI 14.692–374.959, *p* < 0.0001, respectively).

**Table 4:** Logistic regression of echocardiographic parameters predicting cardiac events

	Cardiac events		Univariate			Multivariate (model 1)			Multivariate (model 2)		
	Present (*n* = 21)	Absent (*n* = 1305)	OR	95% CI	*p*-value	OR	95% CI	*p*-value	OR	95% CI	*p*-value
Mitral regurgitation: ≥ moderate, n (%)	5 (24)	79 (6)	4.850	1.732–13.579	0.003						
Tricuspid regurgitation; ≥ moderate, n (%)	11 (52)	101 (8)	13.118	5.438–31.617	< 0.0001	5.248	1.830–14.971	0.004	5.245	1.732–15.797	0.006
Left atrial volume index, ml	63 ± 50	26 ± 22	1.014	1.008–1.024	< 0.001						
Left ventricular end-diastolic volume, ml	134 ± 71	90 ± 33	1.019	1.011–1.026	< 0.0001						
Left ventricular ejection fraction, %	58 ± 19	69 ± 12	0.949	0.924–0.974	< 0.001						
Left ventricular mass index, g/m^2^	124 ± 39	91 ± 26	1.029	1.018–1.039	< 0.0001	1.022	1.009–1.036	0.002	1.023	1.008–1.037	0.001
E/e’	21.1 ± 11.2	11.2 ± 4.2	1.191	1.126–1.265	< 0.0001	1.137	1.069–1.209	< 0.0001	1.131	1.053–1.216	< 0.001
Right atrial pressure: ≥ 15 mmHg, n (%)	4 (19)	7 (0.5)	43.630	11.674–163.058	< 0.0001	18.930	4.104–87.319	< 0.001	NA		
IRVF abnormality, n (%)	7 (33)	6 (0.5)	108.25	32.250 –363.355	< 0.0001	NA			74.223	14.692–374.959	< 0.0001

*CI* confidence interval, *IRVF* intrarenal vein flow, *NA* not applicable, *OR* odds ratio

**Fig. 2:**
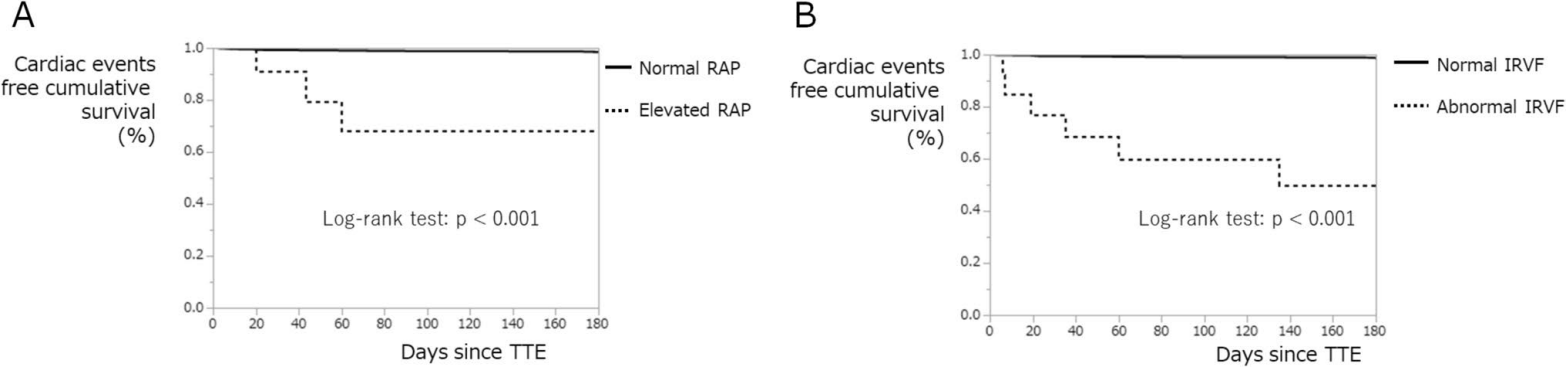
Cumulative survival without cardiac events. **A**, right atrial pressure; **B**, intrarenal vein flow. *IRVF* intrarenal vein flow, *RAP* right atrial pressure, *TTE* transthoracic echocardiography

The Kaplan–Meier plots to predict the occurrence of cardiovascular events are shown in Fig. 2. They reveal the cumulative probabilities of cardiac-free survival within 6 months after the TTE index day. Differences between groups were assessed using the log-rank test. The plots show significantly better outcomes in patients with non-elevated RAP (vs. elevated RAP, *p* < 0.001) and with normal IRVF (vs. abnormal IRVF, *p* < 0.0001).

**Fig. 3:**
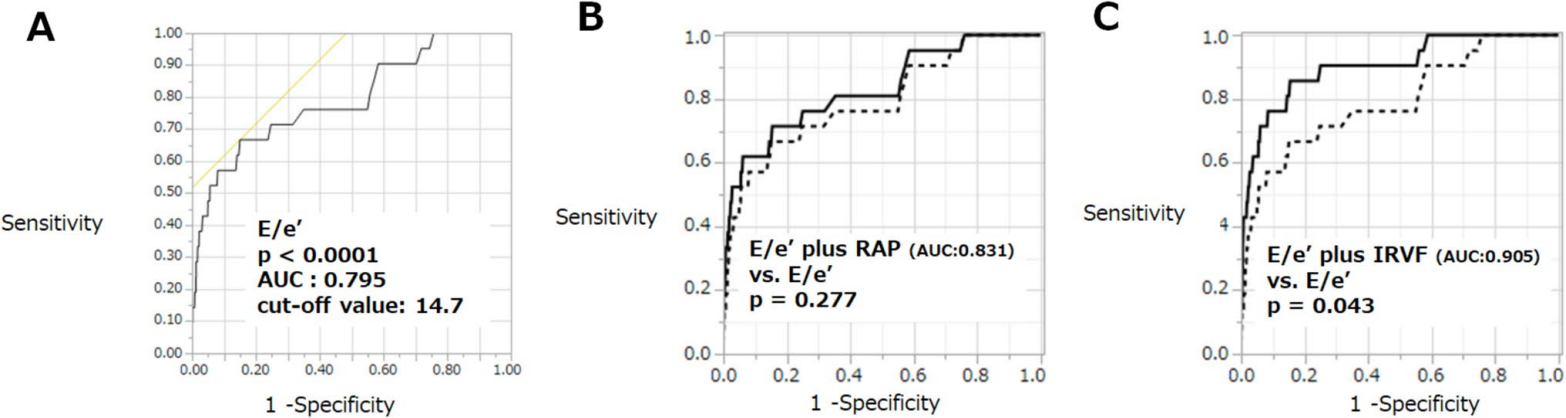
Receiver operating curve analysis to predict the occurrence of cardiovascular events. **A**, E/e’; **B**, E/e’ vs. E/e’ plus right atrial pressure; **C**, E/e’ vs. E/e’ plus intrarenal vein flow. The dashed line indicates the curve for E/e’ in panels **B** and **C**. The solid line indicates the curve for E/e’ plus RAP in panel **B** and the curve for E/e’ plus IRVF in panel **C**. AUC, area under the curve; *IRVF* intrarenal vein flow, *RAP* right atrial pressure

As determined via ROC analysis (Fig. 3A), E/e’ had moderate predictive potential (AUC: 0.795, cutoff value: 14.7, *p* < 0.0001). The Delong test revealed that the combination of E/e’ and IRVF abnormality had better predictive potential than did E/e’ alone (*p* = 0.043), whereas the combination of E/e’ and elevated RAP did not have better predictive value than did E/e’ alone (*p* = 0.277) (Fig. 3B and C).

## Discussion

We prospectively evaluated the utility of brief IRVF scans during routine comprehensive TTE examinations in a standard TTE laboratory. This study has three main findings. First, IRVF patterns were quickly determined in > 50% of the patients in the study cohort. Second, although rare, IRVF abnormalities were associated with cardiac events, especially heart failure, as well as E/e’ and RAP, despite discrepancies between the IRVF and RAP results in a few patients. Third, unlike RAP, IRVF abnormality improved the predictive value of E/e’.

IRVF determined by renovascular ultrasound is a useful tool for detecting visceral congestion, including renal congestion [16, 17]. That IRVF changes are caused by the transition from euvolemia to intravascular volume expansion [18] is the fundamental basis of pathophysiology underlying heart failure. Several reports have emphasized the usefulness of IRVF for predicting outcomes in patients with heart failure [19–22].

We obtained IRVF images from > 50% of the consecutive patients in our study without incurring special costs or requiring additional laboratory resources. Five-minute IRVF scanning was feasible owing to its performance by a single ultrasound technician using the same ultrasound machine as used for TTE, with one-sided probe manipulation of the kidney. For this reason, TTE accompanied by IRVF may be an acceptable method with additional value in most TTE laboratories. We believe that IRVF evaluation may be a potent addition or alternative to CVP estimation in predicting cardiac events despite uncertainties, such as discrepancies between IRVF and RAP.

A discrepancy between the results of IRVF imaging and RAP estimation was observed in 16 patients in our study. Because it is caused by renal dysfunction [5, 6], abnormal IRVF without CVP elevation can occur in patients with renal failure. However, the renal function parameters did not differ between patients with both abnormal IRVF and non-elevated RAP and those without this combination. This likely reflects the absence of patients with deteriorated renal function requiring maintenance hemodialysis in our study cohort. Therefore, we believe that the impact of renal function on the discrepancy was minimal.

Several clinical and pathophysiological factors can be possible to explain this discrepancy. First, the influence of the sympathetic nervous system may be one of them [23]. Because the sympathetic nervous system controls the venous vascular tone, circulatory fluid volume in organs is maintained by both total fluid volume and sympathetic nervous activity [24]. When activated, the sympathetic nervous system worsens renal function [25]. Therefore, increased sympathetic nervous activity in patients with heart failure can cause renal congestion and consequent abnormal IRVF even in those without an increase in total fluid volume. Second, the current method of estimating CVP through observation of the IVC by TTE (measured as RAP) is sometimes problematic, inaccurate [4], and even impossible when the patient is intubated. Third, there may be a different cutoff value of CVP for each patient subgroup depending on medical background such as renal failure, arrhythmias, and valvular heart disease. Thus, although unresolved, complex mechanisms may underlie the discrepancy between IRVF evaluation and RAP estimation.

As our study comprised a small percentage (1%) of patients with IRVF abnormalities in this study targeting all commers in the TTE laboratory, comparing its findings to those of previous studies targeting only heart failure patients is difficult. However, logistic regression analysis showed that IRVF predicted heart failure similarly to established echocardiographic indicators such as E/e’. Moreover, the combination of E/e’ and (not RAP, but) IRVF abnormality had greater predictive value than E/e’ alone.

The present study has some limitations. First, the exclusion of a significant percentage (37%) of the consecutive TTE patients may have caused significant bias. Taking characteristics of the excluded patients into account, we speculate that patients with more advanced non-cardiovascular diseases may have limited acoustic windows because of slender body shapes or limited body positions due to reduced physical mobility and thus are more likely to be excluded from IRVF scans. Second, as no patients were on maintenance hemodialysis owing to institutional limitations, the impact of IRVF abnormalities on renal failure and urinary tract disorders may have been underestimated. Third, our data were obtained from a single-center clinical practice. Owing to the above-mentioned limitations, our results and conclusions may not be generalizable to other institutions. However, our findings may be useful for generating hypotheses and guiding the development of large-scale multicenter studies of patients with renal failure.

## Conclusion

We prospectively evaluated the utility of quick IRVF scans in a standard TTE laboratory. Although IRVF abnormalities are rare in routine comprehensive TTE practice, they improve the ability of E/e’ to predict cardiac events, especially heart failure. Further large-scale prospective studies are required to confirm our findings.

## Data Availability

The datasets generated and analyzed in the current study are not publicly available because of patient privacy concerns.

## References

[1] Damman K, van Deursen VM, Navis G, Voors AA, van Veldhuisen DJ, Hillege H (2009) Increased central venous pressure is associated with impaired renal function and mortality in a broad spectrum of patients with cardiovascular disease. J Am Coll Cardiol 53:582–588 19215832 10.1016/j.jacc.2008.08.080

[2] Mullens W, Abrahams Z, Francis GS, Sokos G, Taylor DO, Starling RC, Young JB, Tang WHW (2009) Importance of venous congestion for worsening of renal function in advanced decompensated heart failure. J Am Coll Cardiol 53:589–596 19215833 10.1016/j.jacc.2008.05.068 PMC2856960

[3] Pellicori P, Carubelli V, Zhang J, Castiello T, Sherwi N, Clark AL, Cleland JG (2013) IVC diameter in patients with chronic heart failure: relationships and prognostic significance. JACC Cardiovasc Imaging 6:16–28 23328557 10.1016/j.jcmg.2012.08.012

[4] Seo Y, Iida N, Yamamoto M, Machino-Ohtsuka T, Ishizu T, Aonuma K (2017) Estimation of central venous pressure using the ratio of short to long diameter from cross-sectional images of the inferior vena cava. J Am Soc Echocardiogr 30:461–467 28065586 10.1016/j.echo.2016.12.002

[5] Bertolotto M, Quaia E, Galli G, Martinoli C, Locatelli M (2000) Color Doppler sonographic appearance of renal perforating vessels in subjects with normal and impaired renal function. J Clin Ultrasound 28:267–266 10867664 10.1002/1097-0096(200007/08)28:6<267::aid-jcu1>3.0.co;2-p

[6] Jeong SH, Jung DC, Kim SH, Kim SH (2011) Renal venous doppler ultrasonography in normal subjects and patients with diabetic nephropathy: value of venous impedance index measurements. J Clin Ultrasound 39:512–518 21544829 10.1002/jcu.20835

[7] Bateman GA, Cuganesan R (2002) Renal vein Doppler sonography of obstructive uropathy. AJR Am J Roentgenol 178:921–925 11906873 10.2214/ajr.178.4.1780921

[8] Oktar SO, Yücel C, Ozdemir H, Karaosmanoglu D (2004) Doppler sonography of renal obstruction: value of venous impedance index measurements. J Ultrasound Med 23:929–936 15292561 10.7863/jum.2004.23.7.929

[9] de la Espriella-Juan R, Núñez E, Miñana G, Sanchis J, Bayés-Genís A, González J, Chorro J, Núñez J (2018) Intrarenal venous flow in cardiorenal syndrome: a shining light into the darkness. ESC Heart Fail 5:1173–1175 30295431 10.1002/ehf2.12362 PMC6300820

[10] Mitchell C, Rahko PS, Blauwet LA, Canaday B, Finstuen JA, Foster MC, Horton K, Ogunyankin KO, Palma RA, Velazquez EJ (2019) Guidelines for performing a comprehensive transthoracic echocardiographic examination in adults: recommendations from the American Society of Echocardiography. J Am Soc Echocardiogr 32:1–64 30282592 10.1016/j.echo.2018.06.004

[11] Nagueh SF, Smiseth OA, Appleton CP, Byrd BF 3rd, Dokainish H, Edvardsen T, Flachskampf FA, Gillebert TC, Klein AL, Lancellotti P, Marino P, Oh JK, Popescu BA, Waggoner AD (2016) Recommendations for the evaluation of left ventricular diastolic function by echocardiography: an update from the American Society of Echocardiography and the European Association of Cardiovascular Imaging. J Am Soc Echocardiogr 29:277–314 27037982 10.1016/j.echo.2016.01.011

[12] Lang RM, Badano LP, Mor-Avi V, Afilalo J, Armstrong A, Ernande L, Flachskampf FA, Foster E, Goldstein SA, Kuznetsova T, Lancellotti P, Muraru D, Picard MH, Rietzschel ER, Rudski L, Spencer KT, Tsang W, Voigt JU (2015) Recommendations for cardiac chamber quantification by echocardiography in adults: an update from the American Society of echocardiography and the European Association of Cardiovascular Imaging. J Am Soc Echocardiogr 28:1–39 25559473 10.1016/j.echo.2014.10.003

[13] Baumgartner H, Hung J, Bermejo J, Chambers JB, Edvardsen T, Goldstein S, Lancellotti P, LeFevre M, Miller F Jr, Otto CM (2017) Recommendations on the echocardiographic assessment of aortic valve stenosis: a focused update from the European association of cardiovascular imaging and the American society of echocardiography. J Am Soc Echocardiogr 30:372–392 28385280 10.1016/j.echo.2017.02.009

[14] Zoghbi WA, Adams D, Bonow RO, Enriquez-Sarano M, Foster E, Grayburn PA, Hahn RT, Han Y, Hung J, Lang RM, Little SH, Shah DJ, Shernan S, Thavendiranathan P, Thomas JD, Weissman NJ (2017) Recommendations for noninvasive evaluation of native valvular regurgitation: a report from the American Society of echocardiography developed in collaboration with the society for cardiovascular magnetic resonance. J Am Soc Echocardiogr 30:303–371 28314623 10.1016/j.echo.2017.01.007

[15] DeLong ER, DeLong DM, Clarke-Pearson DL (1988) Comparing the areas under two or more correlated receiver operating characteristic curves: a nonparametric approach. Biometrics 44:837–845 3203132

[16] Puzzovivo A, Monitillo F, Guida P, Leone M, Rizzo C, Grande D, Ciccone MM, Iacoviello M (2018) Renal venous pattern: a new parameter for predicting prognosis in heart failure outpatients. J Cardiovasc Dev Dis 5:52 30400289 10.3390/jcdd5040052 PMC6306853

[17] Tang WH, Kitai T (2016) Intrarenal venous flow: a window into the congestive kidney failure phenotype of heart failure? JACC Heart Fail 4:683–686 27395345 10.1016/j.jchf.2016.05.009

[18] Nijst P, Martens P, Dupont M, Tang WHW, Mullens W (2017) Intrarenal flow alterations during transition from euvolemia to intravascular volume expansion in heart failure patients. JACC Heart Fail 5:672–681 28711449 10.1016/j.jchf.2017.05.006

[19] Iida N, Seo Y, Sai S, Machino-Ohtsuka T, Yamamoto M, Ishizu T, Kawakami Y, Aonuma K (2016) Clinical implications of intrarenal hemodynamic evaluation by Doppler ultrasonography in heart failure. JACC Heart Fail 4:674–682 27179835 10.1016/j.jchf.2016.03.016

[20] Yoshihisa A, Watanabe K, Sato Y (2020) Intrarenal Doppler ultrasonography reflects hemodynamics and predicts prognosis in patients with heart failure. Sci Rep 10:22257 33335236 10.1038/s41598-020-79351-6 PMC7746684

[21] Yamamoto M, Seo Y, Iida N, Ishizu T, Yamada Y, Nakatsukasa T, Nakagawa D, Kawamatsu N, Sato K, Machino-Ohtsuka T, Aonuma K, Ohte N, Ieda M (2021) Prognostic impact of changes in intrarenal venous flow pattern in patients with heart failure. J Card Fail 27:20–28 32652246 10.1016/j.cardfail.2020.06.016

[22] Kuwata S, Izumo M, Okuno T, Shiokawa N, Sato Y, Koga M, Okuyama K, Tanabe Y, Harada T, Ishibashi Y, Akashi YJ (2004) Impact of renal congestion in patients with secondary mitral regurgitation after mitral transcatheter edge-to-edge repair. Circ J 88:510–516 10.1253/circj.CJ-23-0240 37438144

[23] Ross EA (2012) Congestive renal failure: the pathophysiology and treatment of renal venous hypertension. J Card Fail 18:930–938 23207082 10.1016/j.cardfail.2012.10.010

[24] Fallick C, Sobotka PA, Dunlap ME (2011) Sympathetically mediated changes in capacitance: redistribution of the venous reservoir as a cause of decompensation. Circ Heart Fail 4:669–675 21934091 10.1161/CIRCHEARTFAILURE.111.961789

[25] Firth JD, Raine AE, Ledingham JG (1988) Raised venous pressure: a direct cause of renal sodium retention in oedema? Lancet 1:1033–1035 2896877 10.1016/s0140-6736(88)91851-x

